# Enhanced Uptake of Arsenic Induces Increased Toxicity with Cadmium at Non-Toxic Concentrations on *Caenorhabditis elegans*

**DOI:** 10.3390/toxics10030133

**Published:** 2022-03-10

**Authors:** Chengcheng Pei, Lingyan Sun, Yanan Zhao, Shenyao Ni, Yaguang Nie, Lijun Wu, An Xu

**Affiliations:** 1 Information Materials and Intelligent Sensing Laboratory of Anhui Province, Institutes of Physical Science and Information Technology, Anhui University, Hefei 230601, China; q19301212@stu.ahu.edu.cn (C.P.); q20201028@stu.ahu.edu.cn (L.S.); q19201076@stu.ahu.edu.cn (Y.Z.); q19201032@stu.ahu.edu.cn (S.N.); ljw@ipp.ac.cn (L.W.); 2 Key Laboratory of High Magnetic Field and Ion Beam Physical Biology, Chinese Academy of Sciences; Anhui Province Key Laboratory of Environmental Toxicology and Pollution Control Technology, High Magnetic Field Laboratory, Hefei Institutes of Physical Science, Chinese Academy of Sciences, Hefei 230031, China

**Keywords:** cadmium, arsenic species, joint toxicity, *C. elegans*, bioaccumulation

## Abstract

Cadmium (Cd) and arsenic (As) are widely distributed pollutants that co-exist in the environment; however, their joint toxicity on living organisms is still largely unknown. In this study, we explored the joint toxicity of concurrent exposure to Cd and different As species at low concentrations on *Caenorhabditis elegans* (*C. elegans*) in comparison to single exposures. Endpoints such as germ cell apoptosis, the number of oocytes, brood size, and the life span were employed to evaluate the combined effects of Cd and As on exposed *C. elegans* from L3 or L4 stages. Our results showed that concurrent exposure to non-toxic concentrations of Cd and As caused the synergy of reproductive and developmental toxicity. The presence of Cd promoted the accumulation of As in both germline and intestine detected by laser ablation inductively coupled plasma mass spectrometry (LA-ICP-MS). Although a conversion of As(III) to As(V) was detected as dependent on pH according to the microenvironment of the intestine in the worm, there was no significant difference of toxicity in *C. elegans* concurrently exposed to Cd and different As species. Using loss-of-function mutant strains, As was deemed responsible for the enhanced joint toxicity, and in which *gcs-1* played a key protective role. These data help to better evaluate the comprehensive adverse effects of concurrent exposure of heavy metals at low concentrations on living organisms in the environment.

## 1. Introduction

Heavy metal pollutants are widely distributed in the natural environment due to local geological background and intensified industrialization [1]. Cadmium (Cd) and arsenic (As) receive the most attention due to their threat to the surrounding ecology and human health [2,3]. Both Cd and As have been recognized as carcinogens with high toxicity [4]. Cadmium is well known as the cause of “itai-itai disease” in Japan [5], and trace amounts of Cd entering the human body may lead to kidney, liver, and bone damage [6]. Cadmium decreases DNA polymerase fidelity, inhibits DNA repair [7], and significantly increases the mutation rate in mammalian cells [8]. Injection of CdCl_2_ into mice causes kidney damage and alters testicular sperm expression in mice [9]. It could also affect the motility of *Caenorhabditis elegans* (*C. elegans*) with a significant decrease in crawling speed and bending frequency [10]. Studies showed that Cd inhibited SOD and AChE enzyme activities in *C. elegans*, and caused reproductive and developmental toxicity [11]. Bovio et al. [12] reported that the nematode mortality rose in a dose-dependent manner to Cd concentration due to the loss of enzymatic activity of SOD1. YeonRoh et al. [13] demonstrated that the *mtl-2* (*gk125*) had the lowest LC50 among mutant strains when exposed to Cd, showing significantly less egg production and increased expression of *mtl-2* gene. Arsenic has multiple species with different toxicity in As(III) > As(V) > Dimethylarsinic Acid (DMA) > Monomethylarsonic Acid (MMA) order, indicating a higher risk from inorganic As than organic As [14,15]. It has a significant impact on human health, causing cardiovascular diseases, neurobehavioral disorders, hematological disorders, and various types of diseases [16], by blocking DNA repair and apoptosis, stimulating angiogenesis, causing cell cycle arrest, and inducing aneuploidy [17,18]. Arsenic also threatens various organisms in the environment, inducing tumors in mice, elevating oxidative stress in chicks, and reducing the pigment contents in plants [19,20,21]. Arsenic exposure may cause increased amounts of GST-4, HSP-4, and HSP-70 in *C. elegans*, producing more reactive oxygen species (ROS) and thus causing toxic effects [22]. Yu et al. [23] found that the mRNA levels of transcriptional makers of aging (*hsp-16.1*, *hsp-16.49*, and *hsp-70*) increased in worms under arsenite exposure, accelerating senescence of the nematodes. By parental exposure alone to As, *C. elegans* was found to show decrease in the number of eggs laid in both the parental and offspring generations, and further investigation suggested that the intergenerational reproductive toxicity was due to the decrease of *spr-5* mRNA and the increase of H3K4me2 level in the parental generation [24].

The environmental toxicity of Cd and As is mainly due to their alarming pollution levels in soil and water. The worldwide background value of soil Cd is about 0.35 mg/kg, while the concentration in polluted areas far exceeds this level [25]. In Bangladesh, Cd pollution in water and soil are tens of times higher than the global background value [26]. Cadmium is also considered the most heavily polluted heavy metal in China [26,27,28]. According to the 2014 National Soil Pollution Survey Bulletin, the total exceedance rate of Cd in the soil is 16.1%, of which the highest exceedance rate is 7%, and the exceedance values are tens or even hundreds of times higher than the environmental background value [29]. The world has been exposed to high concentrations of As for a long time [30]. There are more than 10,000 As-contaminated sites in Australia [31]. In Bangladesh, As levels in cultivated soils of Faridpur can reach four times the world limit [27]. In China, the “12th Five-Year Plan for heavy metal pollution control” [32] has recognized arsenic as a first-class heavy metal pollution that needs urgent prevention and control. Arsenic pollution in Xinjiang, Inner Mongolia, Shanxi, and other provinces and regions in China is relatively serious. For example, As concentration in the surface soil of Xi’an reached 14.5 mg/kg, approximately three times more than the world soil background value [33]. As multiple heavy metal pollutants co-exist in the environment, it is hard to reveal the true picture of their hazardous effects by single pollutant exposure, while the joint toxicity of heavy metal exposure urges the need for comprehensive investigation.

The combined toxic effects of two or more compounds on an organism simultaneously or sequentially is defined as joint toxicity [34]. The concurrent exposure effects can be categorized as antagonistic effects, synergistic effects, and additive effects [35,36]. Andrade et al. [37] investigated the neurological and behavioral effects of nickel (Ni) and zinc (Zn) co-exposure on rats, and found that Zn attenuated the motor impairment caused by Ni, indicating antagonistic effects. It was found that co-exposure of mercury (Hg) and Cd inhibited the development of follicles in the human ovary, thereby reducing the number of human oocytes, indicating synergistic effects [38]. Lanier et al. [39] used edible kale and white clover as models to study the chronic toxic effects of Cd and lead (Pb), and the data showed that the concurrent exposure slowed the growth of roots of white clover and caused DNA damage, indicating synergistic effects. Meng et al. [40] found the chlorophyll content of wheat seedlings by co-exposure of Cd and Pb lying between single exposures, indicating an additive effect. Therefore, the different joint toxicity of heavy metals may be influenced by the type of metals involved, the existing form of pollutants, the accumulation of heavy metals in the organisms, and the test models.

*C. elegans* is a nematode ubiquitously distributed in the environment, such as soil and water. It is sensitive to the toxicity of pollutants, and has short life cycle, large number of offspring, transparent body, simple organ composition, clear genetic background, and various mutant strains, showing remarkable advantages as an environment-related model organism [41]. In addition, *C. elegans* genome has 60–80% of human homologs, which may reveal health implication based on its toxicological research [42,43]. It has been widely used in the study of both single and joint toxicity of various heavy metals so far [11,38]. With the newly developed analytical tools such as laser ablation-inductively coupled plasma-mass spectrometry (LA-ICP-MS), we can further explore the accumulation of heavy metals in different organs of *C. elegans*, providing basis for determining the underlying mechanism of possible toxicity [44,45]. In this study, we explored the joint toxicity of Cd and As at low concentrations on *C. elegans*. The accumulation and distribution of heavy metals in *C. elegans*, the transformation of As in the intestinal microenvironment, and the potential mechanism based on mutant strains were explored to explain the co-toxic effects. This study could provide important information on the potential hazards of heavy metal co-exposure in living organisms, and thus laying basis to devise regulations to better manage the hazards of heavy metals.

## 2. Materials and Methods

### 2.1. Preparation of Test Solutions

We used CdCl_2_ (99% purity, AR, Aladdin Group Chemical Reagent Co., Ltd., Shanghai, China) and NaAsO_2_ (>90% purity, AR, Aladdin Group Chemical Reagent Co., Ltd., Shanghai, China) as reference materials to prepare the stock solutions by dissolving precisely weighted powder samples in Milli-Q water to 1 mM. Arsenic(V) oxide hydrate reference material of 1000 mg/L (99%, Inorganic Ventures, Lakewood, NJ, USA) and the stock solutions of CdCl_2_ and NaAsO_2_ were stored in a refrigerator at 4 °C until use. Different working concentrations were prepared by diluting the stock solutions with K-medium [KM, 51 mM NaCl (99.5%purity, AR, Sinopharm Chemical Reagent Co., Ltd., Shanghai, China) + 32 mM KCl (99.5%purity, AR, Sinopharm Chemical Reagent Co., Ltd., Shanghai, China)] before use.

### 2.2. Test Organism

According to the standard procedure described by Brenner [46], all worm strains are cultured on nematode growth medium (NGM) at 20 °C in dark, with *Escherichia coli* (*E. coli*) OP50 seeded as food. Strains including N2 (wild-type Bristol), *mtl-2* (*gk125*), and *gcs-1* (*ok436*) are provided by Caenorhabditis Genetics Center (CGC). Synchronized worms can be obtained when gravid hermaphrodites were lysed in an alkaline hypochlorite solution, and the harvested eggs are hatched overnight at 20 °C without food to remain at L1 stage.

### 2.3. Toxicity Experiments

Germ cell apoptosis: According to the standard procedure described by Gartner [47], apoptotic germ cells were detected by vital staining with acridine orange (AO) (Shenggong Bioengineering Co., Ltd., Shanghai, China). *C. elegans* at L4 stage were exposed to designated working solutions on Costar 24-well plates. After exposure, the solutions containing *C. elegans* were aspirated into centrifuge tubes, and the worms were collected after natural settling, then placed in 400 μL of 75 g/mL AO with OP50 for an incubation at 20 °C for 60 min. After repeated elution, *C. elegans* were moved to NGM plates for a 60 min recovery on bacterial lawns. The purpose of recovery is to remove AO from the intestine of the worms and make it easier for subsequent detection of apoptotic germ cells. The worms were then anaesthetized with sodium azide 2% (NaN_3_) (>99.5%, Sigma-Aldrich, St. Louis, MO, USA) and examined under an Axioscope 5 fluorescence microscope (1769-608, Zeiss, Germany) at 20×. After AO staining, the apoptotic cells were yellow-green, indicating increased DNA fragmentation, while all intact cells were uniformly green. Numbers of apoptotic cells were obtained from at least 20 worms for each group.

Oocyte assay: The *C. elegans* oocyte count was referred to the method of Ruan et al. [48]. Following the same exposure scenario as germ cell apoptosis, 20 treated *C. elegans* from each group were placed on agar plate and anaesthetized with a drop of 2% NaN_3_. The number of oocytes was counted from the spermatheca to the gonad loop per gonad arm under an optical microscope.

Brood-size assay: According to the method of Ruan et al. [48], 15 worms were randomly selected and transferred to 3 mm NGM plates seeded with OP50 after 24 h of exposure from L3 stage. Adults *C. elegans* were transferred to new NGM plates every 24 h until oviposition ceased (approximately 5 days), and eggs laid on the NGM plates were counted under a microscope (K series, Motic, Guangzhou, China).

Life-span assay: According to the method of Du et al. [49], lifespan assays were conducted in 96-well plates containing designated working concentrations, 20 μg/mL 5-fluoro-2′-deoxyuridine (5-FudR, >99%, Sigma-Aldrich, St. Louis, MO, USA) to prevent egg-laying, and concentrated OP50 in KM with a total volume of 200 μL. A total of 20 *C. elegans* grown synchronously to L3 stage were transferred to the plates, and one worm was placed in each well. The *C. elegans* were observed daily under a microscope, and worms with a lack of response to physical stimulation were deemed dead. Survival curves were recorded by continuously exposing *C. elegans* to test solutions until all worms were dead.

### 2.4. Cd and As Accumulation in C. elegans

Synchronized L1 *C. elegans* were placed on NGM plates for a cultivation of 48 h and collected. Since the working concentrations were low, we needed to raise a large number of *C. elegans* for the test. After being exposed in 9 mm Petri dishes for 24 h, *C. elegans* were washed at least 3 times with Milli-Q water in order to remove the exposure solutions on the nematodes. After washing and removing the supernatant, the worms were frozen at −20 °C in a refrigerator and freeze-dried for at least 48 h until constant weight was reached. Dried worms were precisely weighted, then digested in HNO_3_ (99.5%purity, GR, Sinopharm Chemical Reagent Co., Ltd., Shanghai, China) by electric heating at 150 °C. The contents of Cd and As in *C. elegans* were determined by inductively coupled plasma-mass spectrometry (ICP-MS, NexION 2000, Perkin Elmer, Waltham, MA, USA) and atomic fluorescence spectrometer (AFS-930, Titan Instruments, Beijing Jitian Instrument Co., Ltd., Beijing, China), respectively, based on dry weight.

### 2.5. Cd and As Distribution in C. elegans

The distribution of Cd and As in the *C. elegans* was determine by LA-ICP-MS at the Mineral Deposit and Exploration Center (ODEC) of Hefei University of Technology, using a laser ablation system (LA) (Photon Machines Analyte HE with a 193 nm ArF Excimer, Omaha, NE, USA), coupled to Quadrupole-based ICP-MS (Agilent 7900, Santa Clara, CA, USA). L4 worms were treated for 24 h and rinsed three times with Milli-Q water. Randomly selected worms were picked to droplets of Milli-Q water on microscopic slides. Single separated *C. elegans* were obtained after the droplets have been air-dried. The slides were then loaded onto the LA system according to the method of Dai et al. [50], for a scanning with a beam size of 8 μm at a speed of 8 μm/s, laser energy of 2 J/min, laser frequency at 10 Hz, carrier gas He flow rate of 0.9 L/min, and the atomizer Ar flow rate of 0.87 L/min. Mapping images were generated using software lims 5.0.

### 2.6. As Species Alteration Corresponding to pH

We adjusted the pH of KM by HCl and NaOH (95% purity, AR, Sinopharm Chemical Reagent Co., Ltd., Shanghai, China) to 3.6, 4, 5, and 6 to simulate the microenvironment in the intestine of *C. elegans*. After pH adjustment, KM solutions of different pH were stocked in centrifuge tubes and marked. Cadmium, As(III), and As(V) at 1000 μg/mL of 4 μL, respectively, were added to 10 mL KM of different pH and mixed for 24 h. Arsenic species change was detected by atomic fluorescence spectroscopy (LC-AFS) (AFS-930, Beijing Jitian Instrument Co., Ltd., Beijing, China) at Instruments Center for Physical Science, University of Science and Technology of China. The carrier gas flow rate was set at 400 mL/min and the shielding gas flow rate at 600 mL/min. The results were processed by LC-AFS Data Workstation V5.5.3.

### 2.7. Statistical Analysis

All data of toxicity assays in this study are expressed as mean ± standard deviation, based on at least three independent experiments. The difference between groups is analyzed by One-Way ANOVA with Graphpad prism 8, and *p* < 0.05 is considered significant.

## 3. Results

### 3.1. Joint Toxicity of Cd and As at Non-Toxic Concentrations on C. elegans

**Figure 1:**
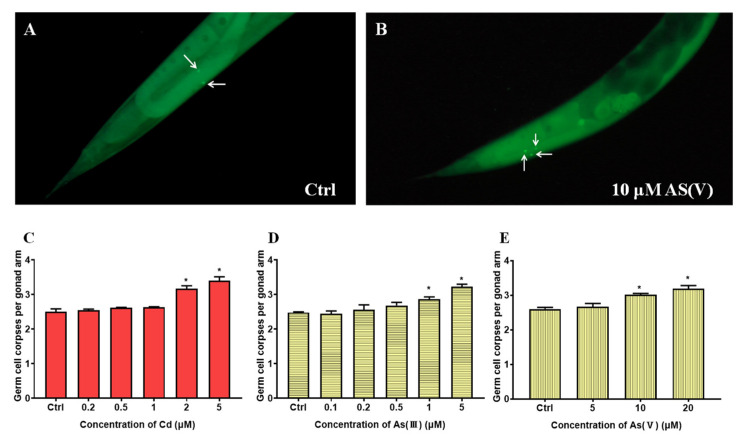
Images of AO stained apoptotic cells in the gonads (**A**,**B**) and the determination of non-toxic concentrations of (**C**) Cd, (**D**) As(III), and (**E**) As(V) based on germ cell apoptosis. * Means *p* < 0.05.

**Figure 2:**
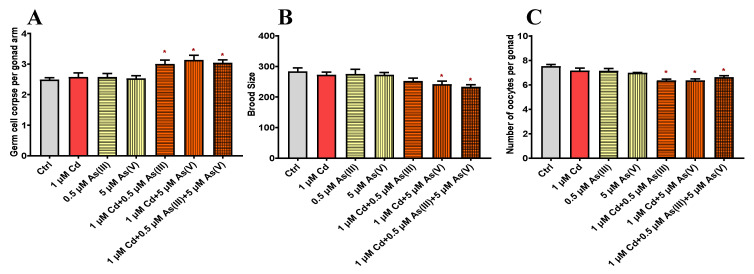
Effects of single and co-exposure to non-toxic concentrations of Cd, As(III), and As(V) on (**A**) germ cell apoptosis, (**B**) brood size, and (**C**) the number of oocytes. * Means *p* < 0.05.

**Figure 3:**
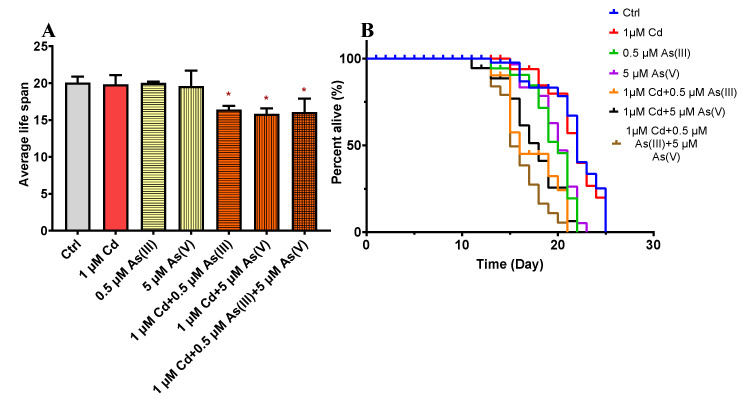
Effects of single and co-exposure to non-toxic concentrations of Cd, As(III), and As(V) on (**A**) average life span and (**B**) survival rate. * Means *p* < 0.05.

The maximum non-toxic concentrations of Cd, As(III), and As(V) are determined based on germ cell apoptosis. As shown in Figure 1, when Cd, As(III), and As(V) reached 2, 1, and 10 μM, respectively, significant differences from control groups were observed, deeming 1, 0.5, and 5 μM as the maximum non-toxic concentrations for Cd, As(III), and As(V), respectively. The following toxicity assays were all carried out using these concentrations. To explore the joint toxicity of Cd and As, the following experiments are carried out on a control group and six test groups including the single exposure of Cd, As(III), and As(V), and co-exposure of Cd-As(III), Cd-As(V), and Cd-As(III)-As(V) at their maximum non-toxic concentrations, respectively. Reproductive toxicity is evaluated by three end points including germ cell apoptosis, brood size, and the number of oocytes. As shown in Figure 2A, the number of apoptotic germ cells increased significantly in co-exposure groups compared to single exposure groups. Brood size and the number of oocytes showed similar results (Figure 2B,C), except that Cd-As(III) co-exposure did not significantly decrease eggs laid (Figure 2B). Co-exposure significantly reduced the lifespan of *C. elegans* to 16.4 ± 0.30 days for Cd-As(III), 15.9 ± 0.43 days for Cd-As(V), and 15.8 ± 1.06 days for Cd-As(III)-As(V) from 20 ± 0.50 days for the control group, in agreement with reproductive toxicity (Figure 3). These data indicated that concurrent exposure to Cd and As caused synergies in both reproductive and development toxicity.

### 3.2. The Accumulation and Distribution of Cd and As in C. elegans

**Figure 4:**
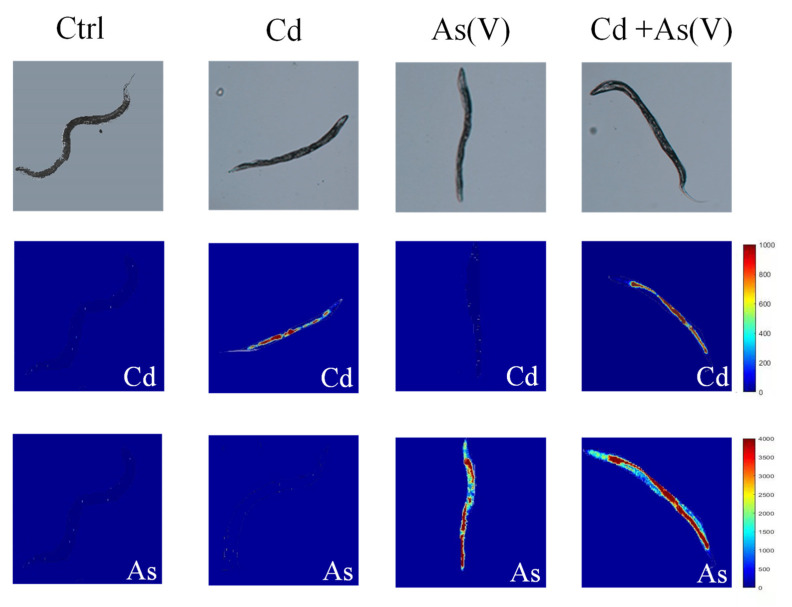
Spatial distribution of Cd and As in *C. elegans* exposed to Cd, As(V), and Cd-As(V).

To explore the mechanism behind the synergies of Cd and As, we analyzed the accumulation of the heavy metals in *C. elegans* using ICP-MS. Since the non-toxic concentrations were too low to carry out measurements on As species, As accumulation was only determined as total As concentrations in Cd-As(III) and Cd-As(V) groups. The results showed that the mean value of As in the single As(III) group was 0.7 ± 0.15 μg/g, and the mean value of As in the Cd-As(III) group was 0.84 ± 0.04 μg/g, about 1.11 times more than the single exposure group. Similarly, As concentration was 310 ± 29.1 μg/g in the single As(V) group, and the mean value of As was 370 ± 31.2 μg/g when Cd and As(V) were co-exposed, which was 1.17 times more than the single exposure group. Cd content in the *C. elegans* did not show significant change in single and concurrent exposure groups. LA-ICP-MS was further employed to explore the distribution of Cd and As in *C. elegans*. Since the non-toxic concentration of As(III) was too low for LA-ICP-MS analysis, the distribution of Cd and As in *C. elegans* was only measured in the Cd-As(V) group (Figure 4). Randomly selected *C. elegans* were scanned, and from the visual result of mapping, we found a higher accumulation of As after co-exposure than that of As alone, and the accumulation was mainly in the intestine and gonad. LA-ICP-MS is a semi-quantitative analysis, the accumulation of As can be calculated based on every pixel of measured worms in relative calorific units. The value of single As(V) exposure was 2017.53 and the value of Cd-As(V) co-exposure was 2739.47, in agreement with ICP-MS results.

### 3.3. The Influence of Intestinal Microenvironment (pH) on As Species

**Figure 5:**
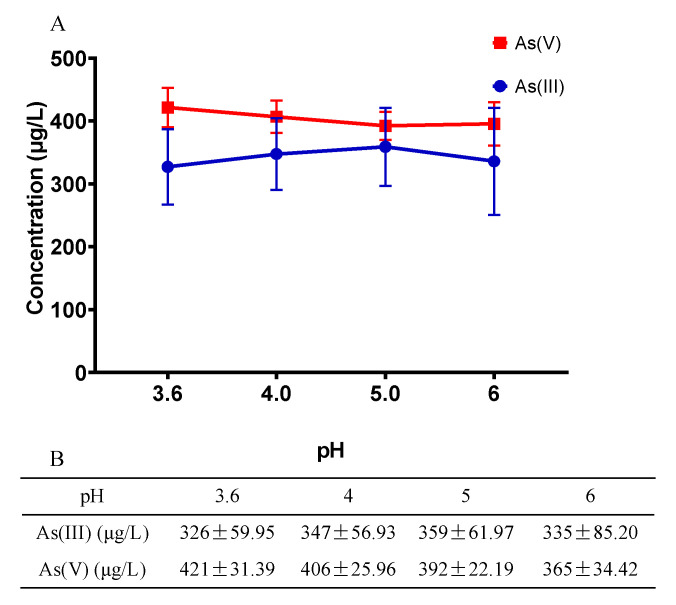
Changes of As species corresponding to different pH. (**A**) As(III) and As(V) trends, (**B**) As(III) and As(V) contents.

Since As has multiple valence states with different toxicity, ingested As may alter in species in the intestine of *C. elegans* due to the microenvironment. Considering the complexity of the intestinal microenvironment, we chose pH here to simulate *C. elegans* intestine, hoping to detect the physicochemical changes of As. The *C. elegans* gut has a pH range from 6 in the anterior pharynx to 3.6 in the posterior gut [44]. We adjusted the pH of the KM solution to 3.6, 4, 5, and 6, and both As species were added for an incubation of 24 h. The concentrations of As(III) and As(V) were determined by LC-AFS. Apart from the data of pH = 6, As(V) was found to increase as pH decreased, while As(III) decreased, indicating part of As(III) was converted to As(V) (Figure 5).

### 3.4. The Effects of mtl-2 and gcs-1 Gene on the Joint Toxicity of Cd and As

**Figure 6:**
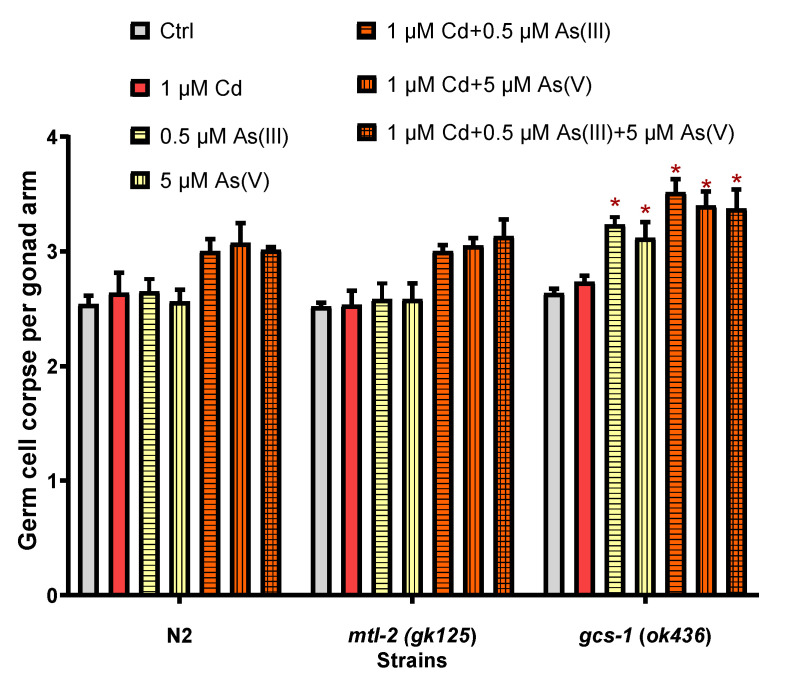
The effect of single and co-exposed Cd, As(III), and As(V) on apoptosis gonad cells of strains *mtl-2* (*gk125*) and *gcs-1* (*ok436*). * Means compared with the corresponding groups of N2, *p* < 0.05.

We observed changes in the amount of germ cell apoptosis by exposing the mutant strains *mlt-2* (*gk125*) and *gcs-1* (*ok436*) to the same working solutions. *mlt-2* (*gk125*) showed no significant change in the amount of germ cell apoptosis with N2, while *gcs-1* (*ok436*) exhibited more germ cell apoptosis in both single and co-exposure groups of As (Figure 6).

## 4. Discussion

To cope with the increasingly serious pollution caused by heavy metals, various managements were conducted, and strict regulations were applied. For example, the content of Cd in rice was limited to less than 0.2 mg/kg, and total arsenic in food was not allowed to exceed 0.6 mg/kg in China [51]. However, pollutants naturally co-exist in the environment, and the extra possible effects derived from the interaction of multiple pollutions at low concentrations should not be overlooked [49,52,53,54]. Therefore, it is environmentally reasonable to explore the combined toxicity of heavy metals at non-toxic levels. The gonad is one of the most important organs in *C. elegans* [50,55], and germ cell apoptosis is a highly sensitive endpoint for evaluating toxicity [51,56]. The mechanism of germ cell apoptosis under Cd and As exposure has been sufficiently discussed in previous studies, showing the dominant role played by the core apoptosis pathway when exposed to As [57,58], and DNA damage-induced apoptosis under Cd exposure [59]. We determined the non-toxic concentrations of Cd, As(III), and As(V) through germ cell apoptosis and conducted the following joint toxicity analysis based on these concentrations. When Cd and As were co-exposed at maximum non-toxic concentrations, the number of apoptotic germ cells was significantly increased compared to the single exposure groups (Figure 2A). Brood size, the number of oocytes (Figure 2B,C), and the lifespan of the worms (Figure 3) showed similar results, indicating that the co-exposure caused synergies in both reproductive and development toxicity. Similar observation on the synergic effects of Cd and As was reported in wheat and *B. pilosa L* [60,61]. Driessnack et al. [62] found that co-exposure to Cuprum (Cu) and Ni damaged the ovaries of fish and caused reproductive toxicity, and further studies revealed that co-exposure increased the accumulation of Cu and Ni in the body. Cadmium and As co-exposure resulted in greater joint toxicity on wheat seedling growth than when Cd and As were exposed alone, increasing the accumulation of Cd and As in vivo [63]. Tremaroli et al. [64] found that the transformation of As species in mice after the entry of As into the body generated different toxic effects. Thus, the synergistic effect may be attributed to the increase in the accumulation of heavy metals after co-exposure and the transformation of heavy metals in the organisms. We then analyzed the accumulation and distribution of Cd and As in *C. elegans* and the possible transformation of As species in the intestine, expecting to explain the synergistic effect through physicochemical mechanisms.

The accumulation and distribution of heavy metals in organisms is the basis for their subsequent toxicity, and different accumulation of heavy metals can lead to different effects [65]. Our results showed that As content in *C. elegans* after the compound exposure was elevated compared to the content in the single exposure groups, while Cd accumulation remained the same, indicating the presence of Cd promoted the accumulation of As in *C. elegans*. Száková et al. [66] reported that co-exposure to Cd and As significantly inhibited body weight gain in rats compared to the single exposure group, and As added to the diet promoted the accumulation of Cd in the testes. Cao et al. [61] found a significant increase in As accumulation in wheat after combined exposure to Cd and As compared to single exposure. The distribution of Cd and As in *C. elegans* was further obtained by LA-ICP-MS, a method that allows in situ observation of the distribution of heavy metals in organisms with high resolution [67], which has been successfully applied to *C. elegans* [45,68]. Our results showed that As accumulated mainly in the intestine as well as in the gonads of *C. elegans*, and the accumulation in selected nematodes scanned by LA-ICP-MS showed the same trend as the total accumulation measured by ICP-MS, further demonstrating that Cd promoted the uptake of As by *C. elegans* (Figure 4). Arsenic exposure can produce increased germ cell apoptosis due to oxidative stress [69]. Arsenite promotes the production of ROS and lipofuscin accumulation in the *C. elegans* intestine, which causes damage to the digestive tract [24,70]. When Cd and As were exposed in combination, the enhanced As accumulation in the gonad and intestine could be the cause for the synergistic effect.

The intestine is the first biological barrier against exogenous pollutants, whose microenvironment may influence the effects of heavy metals [71,72]. As one of the main target organs of As accumulation in *C. elegans* indicated by LA-ICP-MS, the pH of the intestinal lumen of *C. elegans* ranged from 6 in the anterior pharynx to 3.6 in the end of the intestine [56]. When As enters the body, the metabolic pathway of As(III) is converted to As(V) or methylation, while pH may be one of the important factors affecting As species [73]. Our results showed that as the pH decreased along the intestine, part of As(III) converted to the less toxic As(V) (Figure 5). Previous studies have reported higher toxicity caused by As(III) than As(V) in various test organisms [74,75]. Although As undergoes transformation in the intestine, there is no significant difference between different species of As when exposed with Cd. This is probably due to the fact that although As species conversion was detected, the magnitude is not enough to cause a distinguishable difference in toxicity.

We further investigated the potential mechanism of synergistic effects by the *gk125* mutant strain (*mtl-2* metallothionein gene defective type) and *ok436* mutant strain (*gcs-1* gene defective type). The main physiological function of metallothionein is involved in detoxification of heavy metals in organisms, including Cd [15]. Thus, *mtl-2* in *C. elegans* also plays an important role in protecting against toxic effects caused by Cd detoxification [76]. Wang et al. [58] found that Cd activates caspases through JNK and p38 MAPK signaling pathways, leading to an increased amount of germ cell apoptosis in *C. elegans*. Cadmium exposure causes inflammatory responses and cellular damage in the human intestine, dysbiosis of intestinal flora, and damage to the intestinal tract [77]. Wang et al. [78] reported that *pcs-1* (*mt1748*) exhibited a higher amount of apoptotic germ cells compared to N2 when exposed to the same level of Cd, indicating that the *pcs-1* gene is involved in the toxic regulation of Cd in *C. elegans*. No difference was found in the amount of germ cell apoptosis between *mtl-2* (*gk125*) and N2, and this is likely due to the similar Cd accumulation at a non-toxic level in single and co-exposure with As. It also suggests that even enhanced As accumulation does not respond to *mtl-2*. Wang et al. [22] found insulin-like growth factor-1 (IGF-1) networks and their target protein DAF-16/FOXO, known as key regulators of energy metabolism and growth, played important roles in arsenite-induced apoptosis. Glutathione (GSH) is also known to be an important endogenous antioxidant against exogenous contaminants, interfering with toxic substances by altering the rate of metal uptake as well as clearance, thus protecting cells from metal toxicity [76]. Arsenic readily reacts with thiol-containing molecules (GSH, Cys) [79], and intracellular GSH protects cells from arsenite-induced cytotoxicity in vitro [80,81,82]. Yu et al. [83] found that the *gcs-1* gene is essential for GSH synthesis in *C. elegans* cells, so we used the *gcs-1* gene defective phenotype to study the toxicity caused by As after co-exposure. It was found that when *gcs-1* (*ok436*) was exposed to Cd alone, there was no significant change in the amount of germ cell apoptosis, showing that non-toxic concentration of Cd does not respond to the *gcs-1* gene. After exposure to As(III) or As(V) alone, the amount of germ cell apoptosis in *gcs-1* (*ok436*) increased significantly compared to N2, indicating when *gcs-1* was defective in vivo, the worm will be unable to synthesize GSH to protect against the toxic effects caused by As, resulting in increased toxicity. Thus, we believe the higher joint toxicity of Cd-As co-exposure in *gcs-1* (*ok436*) resulted from the increased accumulation of As in vivo. Therefore, the synergistic effect of Cd-As co-exposure was mainly caused by the increased toxicity of As (Figure 6).

## 5. Conclusions

Our results showed a synergistic effect of Cd and As at non-toxic concentrations on the reproduction and development of *C. elegans*. It was revealed that Cd promoted As uptake by *C. elegans*, increasing As accumulation in the gonad and intestine. Our simulation experiments indicated that the decrease of pH in the intestine may result in the conversion of As(III) to As(V) along the digestive tract, but was not enough to cause a significant difference in joint toxicity. We found that germ cell apoptosis in the *mtl-2* (*gk125*) mutant strain was not significantly different from N2, while *gcs-1* (*ok439*) showed higher reproductive damage, confirming that increased As toxicity dominated the synergistic effect of Cd and As. However, due to the low concentration of exposed As(III) and lack of extraction method, As(III) and As(V) cannot be distinguished concurrently in *C. elegans*, forcing us to rely on the simulation based on the microenvironment of the worm’s intestine. We plan to develop a proper extraction method for As species especially for biological samples, or introduce other in situ detection means such as μ-XANES to address the possible transformation of As(III) and As(V) in vivo. In addition, although the roles of Cd and As in the joint toxicity were determined based on mutant strain studies, the underlying toxicological mechanisms were not fully elucidated. Further exploration focusing on the molecular mechanisms will be carried out in the future. This study indicates co-exposure to heavy metals at non-toxic levels is a potential threat to the ecosystem, which needs further evaluation of other heavy metal combinations and systematical research on the mechanisms involved.

## Data Availability

Not applicable.
